# Regulation of Glioblastoma Progression by Cord Blood Stem Cells Is Mediated by Downregulation of Cyclin D1

**DOI:** 10.1371/journal.pone.0018017

**Published:** 2011-03-24

**Authors:** Kiran Kumar Velpula, Venkata Ramesh Dasari, Andrew J. Tsung, Christopher S. Gondi, Jeffrey D. Klopfenstein, Sanjeeva Mohanam, Jasti S. Rao

**Affiliations:** 1 Department of Cancer Biology and Pharmacology, University of Illinois College of Medicine at Peoria, Peoria, Illinois, United States of America; 2 Department of Neurosurgery, University of Illinois College of Medicine at Peoria, Peoria, Illinois, United States of America; The University of Chicago, United States of America

## Abstract

**Background:**

The normal progression of the cell cycle requires sequential expression of cyclins. Rapid induction of cyclin D1 and its associated binding with cyclin-dependent kinases, in the presence or absence of mitogenic signals, often is considered a rate-limiting step during cell cycle progression through the G_1_ phase.

**Methodology/Principal Findings:**

In the present study, human umbilical cord blood stem cells (hUCBSC) in co-cultures with glioblastoma cells (U251 and 5310) not only induced G_0_-G_1_ phase arrest, but also reduced the number of cells at S and G_2_-M phases of cell cycle. Cell cycle regulatory proteins showed decreased expression levels upon treatment with hUCBSC as revealed by Western and FACS analyses. Inhibition of cyclin D1 activity by hUCBSC treatment is sufficient to abolish the expression levels of Cdk 4, Cdk 6, cyclin B1, β-Catenin levels. Our immuno precipitation experiments present evidence that, treatment of glioma cells with hUCBSC leads to the arrest of cell-cycle progression through inactivation of both cyclin D1/Cdk 4 and cyclin D1/Cdk 6 complexes. It is observed that hUCBSC, when co-cultured with glioma cells, caused an increased G_0_-G_1_ phase despite the reduction of G_0_-G_1_ regulatory proteins cyclin D1 and Cdk 4. We found that this reduction of G_0_-G_1_ regulatory proteins, cyclin D1 and Cdk 4 may be in part compensated by the expression of cyclin E1, when co-cultured with hUCBSC. Co-localization experiments under *in vivo* conditions in nude mice brain xenografts with cyclin D1 and CD81 antibodies demonstrated, decreased expression of cyclin D1 in the presence of hUCBSC.

**Conclusions/Significance:**

This paper elucidates a model to regulate glioma cell cycle progression in which hUCBSC acts to control cyclin D1 induction and in concert its partner kinases, Cdk 4 and Cdk 6 by mediating cell cycle arrest at G_0_-G_1_ phase.

## Introduction

Glioblastoma multiforme (GBM) is the most virulent and fatal form of brain cancer. Currently, no ideal treatment exists for glioblastoma multiforme, and patients generally survive less than one year [1], [2]. Although there have been many achievements in surgery, radiotherapy and chemotherapy, glioblastoma continues to have a very poor prognosis [3]–[5]. Since finding an effective mechanism to deliver therapeutic agents to the targeted site of the tumor has proved problematic, the therapeutic potential of novel gene therapies has been greatly diminished [6].

Several researchers have studied the effect of neuronal stem cells with regard to the tropism and growth reduction of tumors, but their clinical application is restricted by their potential immunologic incompatibility [7]–[10]. Because of these limitations, most studies have focused on evaluating murine neural stem cells. The inherent problems with neural stem cells create the need for evaluation of other types of stem cells that are more readily available and clinically pertinent and may be used as vehicles for delivering therapeutic agents to brain tumors. Human umbilical cord blood (hUCB), a rich source of hematopoietic and mesenchymal stem cells provides an alternative source of stem cells. Of the various progenitor cells that exist in cord blood, mesenchymal stem cells in particular are attractive for clinical use because of their easy isolation, availability, and expansion in cultures [11], [12]. In addition, it has been established that hUCB-derived stem cells (hUCBSC) exhibit higher proliferation and expansion potential than their adult bone marrow counterparts [13], [14]. Recent investigations on mesenchymal stem cells have revealed their participation in tumor growth and metastasis, partially due to their immunosuppressive and proangiogenic properties [15]. Lu *et al*. reported that mesenchymal stem cells could upregulate the expression of p21, a cell cycle inhibitor, at the transcriptional level, thereby facilitating G_0_-G_1_ phase arrest and leading to apoptotic death of the tumor cells [16].

Normal progression of the cell cycle requires sequential expression of cyclins. Cyclins activate cyclin-dependent kinases (Cdk), which comprise a family of serine/threonine protein kinases, to phosphorylate target proteins required for cell-cycle progression [17]. Rapid induction of cyclin D1 and its associated binding with Cdk 4/Cdk 6, in the presence or absence of mitogenic signals, is considered to be a rate-limiting step, and is essential for a cell to pass through G_1_ phase during cell cycle progression. The loss of regulatory control of the cell cycle, which leads to unrestrained cell proliferation, is a hallmark of cancer. Cyclin D1 is overexpressed in breast, liver, lung and brain cancers [18]–[20]. Suppression of cyclin D1 gene expression is an indication of cell differentiation [21]–[23]. Dormant cells, when mitogen stimulated, enter the cell division cycle by activating cyclin D1, along with the dependant cyclin kinases Cdk 4 and Cdk 6, by phosphorylating retinoblastoma protein to release E_2_F transcription factors [24]. In the present study, we sought to provide insight into the functional and regulatory characteristics of cyclin D1, to determine its potential as a therapeutic target for glioblastoma, and to evaluate whether the expression of cyclin D1 is associated with clinical and pathological features in glioblastoma. Here, we show that human umbilical cord blood stem cells (hUCBSC) co-cultured with U251 and 5310 cells resulted in G_0_-G_1_ phase arrest, probably by down regulating the expression of cyclin D1 and its associated kinases Cdk 4 and Cdk 6.

## Results

### hUCBSC arrest cell cycle of glioma cell lines U251 and 5310 at G_0_-G_1_ phase

**Figure 1:**
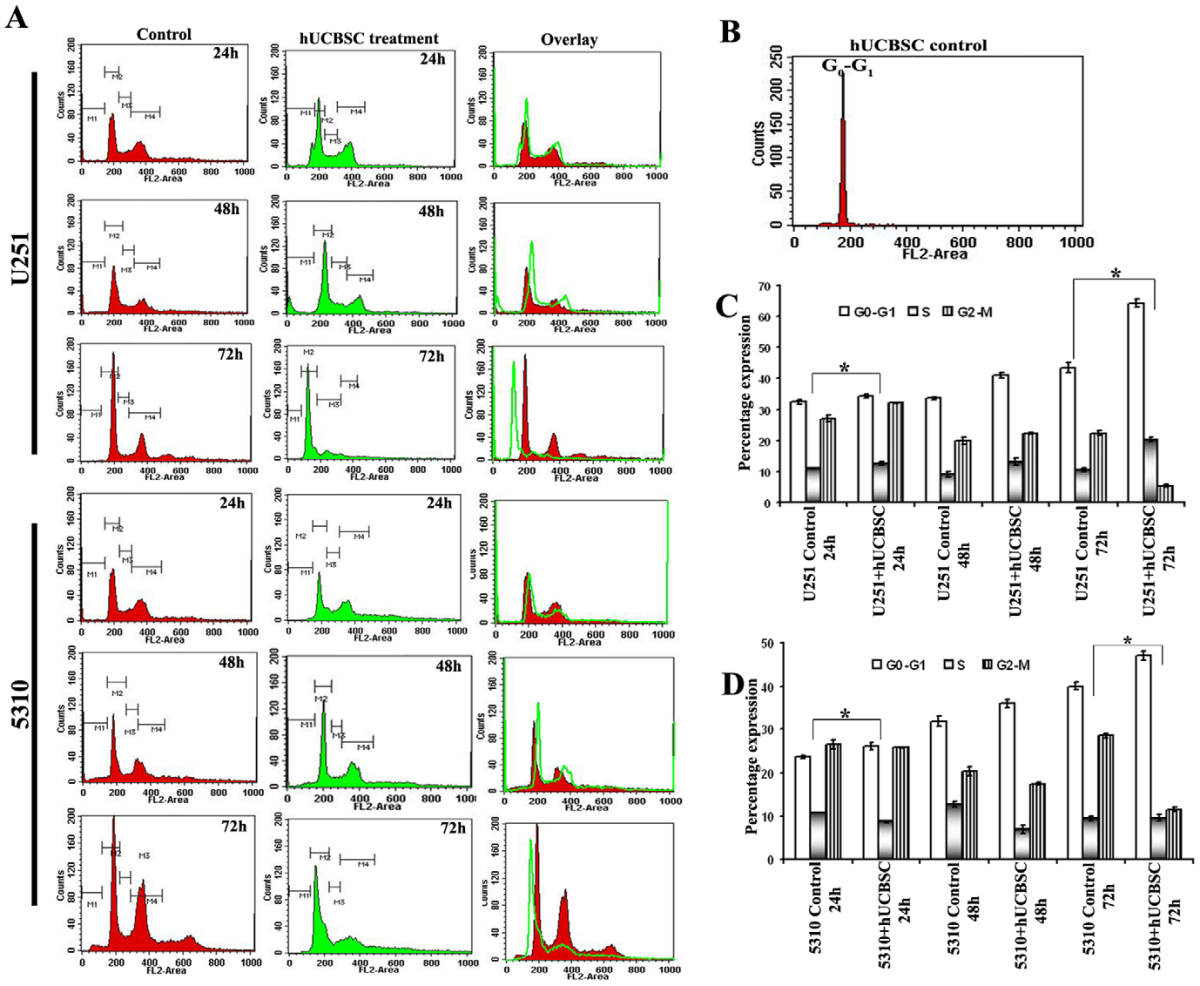
Effect of hUCBSC on cell cycle progression of glioma cells U251 and 5310. (A) Approximately 1×10^6^ cells of U251 and 5310 cells were co-cultured with hUCBSC. Samples were harvested for every 24 h till 72 h to study the percentage of different phases of glioma cell cycle upon hUCBSC treatment. Adherent and detached cells were harvested, pooled, washed once in phosphate-buffered saline (PBS). Cells were treated with propidium iodide at a concentration of 50 µg/ml. After incubation at room temperature for 10–20 min, cells were analyzed for cell cycle distribution with a FACS Calibur flow cytometer (fluorescence-activated cell sorter; FACS) and CellQuest software (Becton Dickinson). Cells with DNA content between 2N and 4N were designated as being in the G_1_, S, or G_2_/M phase of the cell cycle. The number of cells in each compartment of the cell cycle was expressed as a percentage of the total number of cells present. Data shown was obtained from three independent experiments before calculating means and standard deviations. M1, M2, M3 and M4 represent the apoptosis, G_0_-G_1,_ S and G_2_-M phases of the cell cycle respectively. (**B**) hUCBSC were trypsinized, harvested and washed with two volumes of PBS and were processed for FACS analysis as mentioned above. Bar graph representing the kinetics and different phases of cell cycle at different time points in U251 (**C**), 5310 (**D**) and hUCBSC alone and in co-culture. *Significant at p<0.05. n≥3.

The authenticity of hUCBSC was characterized by the expression levels of the specific markers CD29 and CD81. hUCBSC were treated with either CD29 (Fig. S1A) or CD 81 (Fig. S1B) for 2h. They were probed with anti-mouse (green) and anti-goat (green) Alexa fluor secondary antibodies and were subjected to FACS analysis. CD29 expressed 96% whereas CD81 expressed 86% when compared to the controls. Our experiments, designed to determine the role hUCBSC in cancer cell proliferation, were carried out by co-cultures with U251 and 5310 cells. For co-culture experiments, hUCBSC and glioma cells U251 and 5310 were cultured at a ratio of 1∶4. The kinetics of hUCBSC-induced cell cycle arrest was determined by flow cytometric analysis of propidium iodide labeled nuclei. Cells started to accumulate in the G_0_-G_1_ phase of the cell cycle between 24 and 48 h of hUCBSC treatment, and the percentage of cells in the G_0_-G_1_ phase continued to increase after 72 h of co-culture. To further analyze the kinetics of hUCBSC-induced differentiation and proliferation, different phases of the cell cycle were determined by FACS analysis after 24, 48, and 72 h (Fig. 1A). U251 and 5310 control cells after 24 h showed 32%, 24% of G_0_-G_1_ phase, 11%, 10.8% of S phase and 27%, 27% of G_2_-M phases, respectively while hUCBSC treated cells has shown 35%, 26% of G_0_-G_1_ phase, 12%, 9% of S phase and 32%, 25.7% of G_2_-M phase. After 48 h, U251 and 5310 control cells showed 34%, 32% of G_0_-G_1_ phase, 9%, 12.6% of S phase and 20%, 20.4% of G_2_-M phase, while hUCBSC treated cells have shown 41%, 36% of G_0_-G_1_ phase, 13%, 9% of S phase and 22%, 17.46% of G_2_-M phase respectively. hUCBSC co-cultured cells with U251 and 5310 cells analyzed after 72 h showed 64%, 48% of G_0_-G_1_ phase, 20%, 10% of S phase and 5%, 11% of G_2_-M phase, while U251 and 5310 controls have shown 43%, 40% of G_0_-G_1_ phase, 10.5%, 10% of S phase and 22%, 28.58% of G_2_-M phase respectively (Figs. 1C and 1D). However, hUCBSC alone have shown 90–95% of G_0_-G_1_ phase, 2–4% S phase and negligible G_2_-M phase (Fig. 1B).

In order to analyze two different populations of glioma cells and hUCBSC in co-cultures, we used flow cytometry using two different markers, namely CD81 for hUCBSC and GFAP for glioma cells. Data were analyzed using Cell Quest software (BD Biosciences, San Jose, CA). Using forward and side scatter alone did not differentiate the two different cell populations. Plotting CD81-green on the Y-axis versus forward scatter on the X-axis identified two distinct populations of cells. We gated the lower population as R1 and the upper population as R2. Both populations showed green fluorescence in excess of background, however, the upper population showed a much brighter intensity. The R1 population was green only, but the R2 population consists of both red and green cells (as observed in the fluorescent microscope) and contained glioblastoma cells surrounded by multiple hUCBSCs. The increased intensity in green staining may be due to the fact that multiple hUCBSC are surrounding glioma cells. Using this approach, we were able to clearly distinguish U251 and hUCBSC cells (Fig. S2) and 5310 and hUCBSC (Fig. S3) in co-cultures.

### hUCBSC down regulates the expression of cell cycle proteins cyclin D1, Cdk 4 and Cdk 6

**Figure 2:**
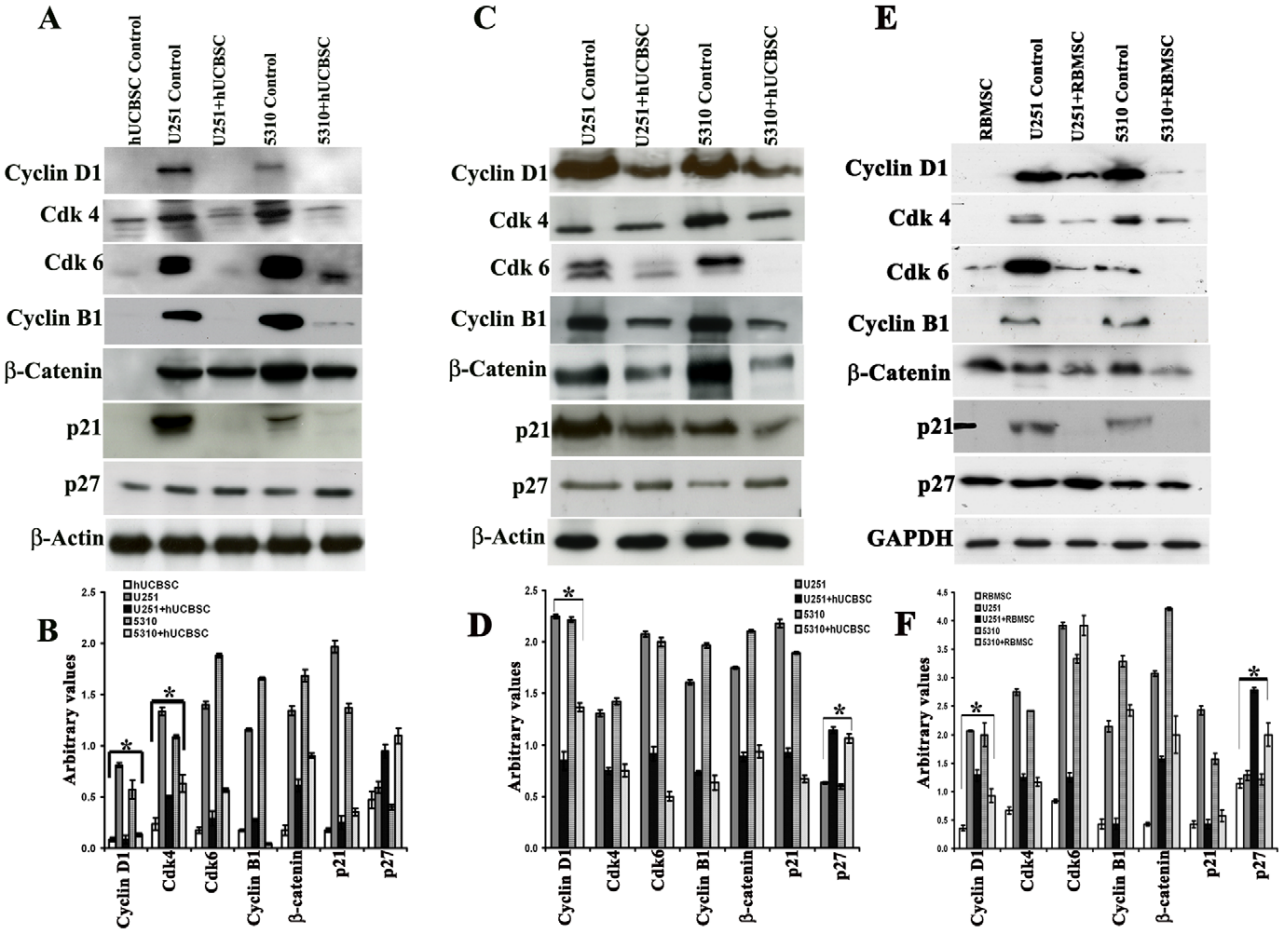
Effect of co-culturing hUCBSC on the expression of various cell cycle proteins and their upstream molecules in U251 and 5310 cells. (**A**) Western blotting of *in vitro* and *in vivo* samples. Briefly, cell lysates from *in vitro* samples (1×10^6^ cells) were prepared from U251 and 5310 cells alone and in co-culture with hUCBSC after 72 h. Cell lysates were prepared as described previously. Western blotting was carried out to determine the effect of hUCBSC on cyclin D1, cyclin B1, Cdk 4, Cdk 6, upstream molecules like β-Catenin and other cell cycle regulatory proteins like p21 and p27. (B) Quantitative estimation of figure A. (**C**) *In vivo* expression was studied by loading equal amounts of protein (40 µg) from tissue lysates of untreated and treated mice brains onto 12% SDS PAGE gels. Around 40 µg of total soluble protein were loaded onto 12% SDS PAGE gels. The transferred proteins were then probed with respective antibodies. β-actin is served as the loading control. Each experiment was repeated 3 times. (D) Quantitative estimation of figure C. (**E**) Single and co-cultures of glioma cells with rat bone marrow stromal cells. ∼40 mg of total protein lysate were loaded onto 12% gels and transferred onto nitrocellulose membranes and probed with respective antibodies. Immuno reactive bands were visualized using chemiluminescence ECL western blotting detection reagents and the reaction was detected using Hyperfilm-MP autoradiography film. GAPDH is served as the loading control. Each experiment was repeated three times. (F) Quantitative data of figure E. *Significant at p<0.05. n≥3.

Analysis of single and co-cultured samples by flow cytometry showed that cells undergo a G_1_ arrest as evident from an increase in the G_0_-G_1_/S ratio, relative to the control cells. Cell cycle progression is controlled by cyclins and cyclin-dependent kinases. To determine the expression of cyclins during G_0_-G_1_ arrest in U251 and 5310 cells after hUCBSC treatments, immunoblotting experiments were done. Western blot analysis confirmed a sharp decrease in both cyclin D1, Cdk 4 and Cdk 6 levels, apart from other cell cycle proteins like cyclin B1 and p21, after 72 h of hUCBSC treatment, when compared with control cells, U251 and 5310 (Fig. 2A). Western blot analysis also showed an increase in p27 protein (Figs. 2A and 2B), coinciding with cell cycle arrest.

To study the effect of hUCBSC on upstream molecules regulating cyclin D1 expression, we screened the expression levels of β-Catenin, and the results indicate that hUCBSC reduced its expression (Figs. 2A, 2B). The results obtained support our hypothesis that the stem cells regulate the expression of cyclin D1, which is an important regulator for the cells to progress in G_0_-G_1_. Similar results were observed when we analyzed cell cycle proteins using tissue lysates (Fig. 2B). Apart from these proteins, we also found the expression of upstream molecules such as GSK3-β, ERK and pERK from tissue lysates treated with hUCBSC to be reduced, when compared with their respective controls (Fig. S4A). To study whether this particular phenomenon of arresting the glioma cells at G_0_-G_1_ when co-cultured was exhibited only by hUCBSC or even other mesenchymal stem cells, we co-cultured rat bone marrow stromal stem cells with U251 and 5310 cell lines for 72 h. A similar type of effect was observed when compared to hUCBSC. Cyclin D1, Cdk 4, Cdk 6, cyclin B1, β-catenin and p21 showed decreased levels of expression, whereas p27 showed increased expression levels (Fig. 2E). We also co-cultured glioma cells with normal human astrocytes and to study the effect of astrocytes on glioma cells. Astrocytes did not show any effect on the expression of cyclin D1, Cdk 4, Cdk 6 and other cell cycle proteins tested, suggesting that hUCBSC indeed regulates the cell cycle progression by down regulating cell cycle proteins (Fig. S4B).

**Figure 3:**
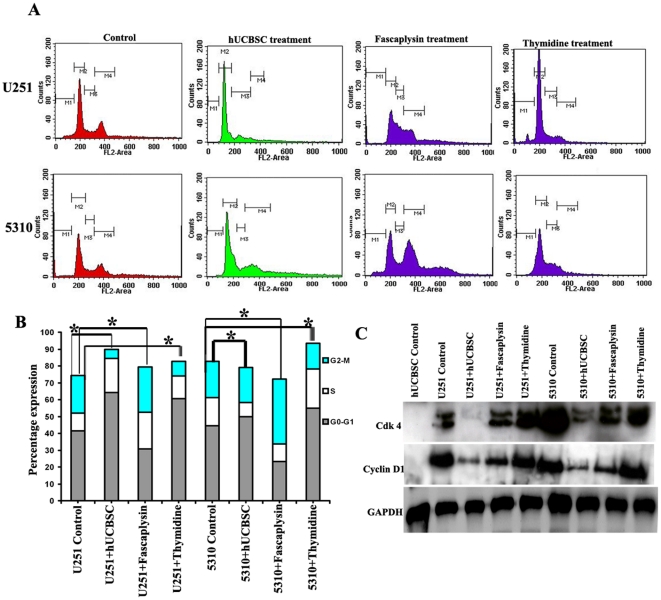
Effect of Fascaplysin and thymidine treatment on glioma cell cycle. (**A**) Cell cycle analysis was done to visualize and study the different phases of cell cycle observed when U251 and 5310 cells were co-cultured with hUCBSC. Control and co-cultured cells were harvested and stained. The data depicted here was collected from 10000 events. FACS analysis was done to assess fascaplysin reduction of various cell cycle phases in U251 and 5310 cells, treated for 72 h with 1 µM fascaplysin. Synchronous cell cycle progression of G1-synchronized cells was studied using 4 mM thymidine. Cells were harvested, fixed and stained with propidium iodide. Cells were observed to be synchronized in the G_0_-G_1_ phase. The G_1_ and G_2_M populations marked were visualized after staining with propidium iodide. M1, M2, M3 and M4 represent the apoptosis, G_0_-G_1,_ S and G_2_-M phases of the cell cycle respectively. (**B**) Bar graph indicating the cell cycle distributions of U251 and 5310 cells, when treated with 1.0 µM fascaplysin, 4.0 mM thymidine in separate experiments were analyzed by FACS. *Significant at p<0.05. (**C**) U251 and 5310 cells were treated for 24 h with 1 µM fascaplysin. The cyclin D1 and Cdk 4 levels were analyzed by western blotting. In another experiment, the glioma cells U251 and 5310 were treated with 4.0 mM thymidine and the cell lysates were analyzed for the presence of cyclin D1 and Cdk 4 by using Western blotting with the indicated antibodies. GAPDH was used as a loading control.

Western blot and FACS analysis have indicated that stem cells may regulate glioblastoma progression by regulating the expression cyclin D1 and its associated kinases Cdk 4 and Cdk 6 (Fig. 2). In order to evaluate this mechanism, we used 1 µM Fascaplysin, a potent inhibitor specific for the cyclin D1/Cdk 4 complex. After 24 h, FACS analysis shows a reduced G_0_-G_1_ peak, indicating that the expression of cyclin D1 and Cdk 4 is necessary for the cells to progress in that phase (Fig. 3A). U251 and 5310 control cells after 24 h showed 42%, 44% of G_0_-G_1_ phase, 10%, 16% of S phase and 22%, 21.43% of G_2_-M phases, respectively while Fascaplysin treated cells has shown 30%, 23% of G_0_-G_1_ phase, 21%, 10% of S phase and 26%, 38.5% of G_2_-M phase (Figs. 3A and 3B). Western blot analysis carried out to study this effect at the protein level indicated a decrease in the expression of Cdk 4 and cyclin D1 (Fig. 3C). In another experiment, we synchronized the glioma cells in G_0_-G_1_ phase using 4 mM thymidine for 12 h in order to study the expression of cyclin D1 and Cdk 4 in the G_0_-G_1_ phase. FACS analyses indicate that the cells treated with 4 mM thymidine were synchronized at the G_0_-G_1_ phase by demonstrating 60%, 55% and 13%, 23% of S phase with 8%, 15% of G_2_-M phases, respectively in both U251 and 5310 cell lines (Fig. 3B). Western blot analysis showed the increased expression of Cdk 4 and cyclin D1 (Fig. 3C) in the thymidine treated cells coinciding with FACS analysis. Fascaplysin treatment indicated reduced expression of cyclin D1 and Cdk 4 and a reduced G_0_-G_1_ whereas when cells were treated with thymidine, the cells showed an increase in G_0_-G_1_ along with increased cyclin D1 and Cdk 4 expression levels. hUCBSC when co-cultured with glioma cells depicts both fascaplysin and thymidine activity in part, by showing reduced G_0_-G_1_ phase, reduced cyclin D1 and Cdk 4 expression levels, and increased G_0_-G_1_ phases with decrease in the cyclin D1 and Cdk 4 expression levels. We thus focused our studies on elucidating the mechanism by which the hUCBSC co-cultures resulted in an increased G_0_-G_1_ phase despite a reduction of G_0_-G_1_ regulatory proteins cyclin D1 and Cdk 4.

### Analysis of expression of cyclin D1 and cyclin E by flow cytometry and immuno precipitation

**Figure 4:**
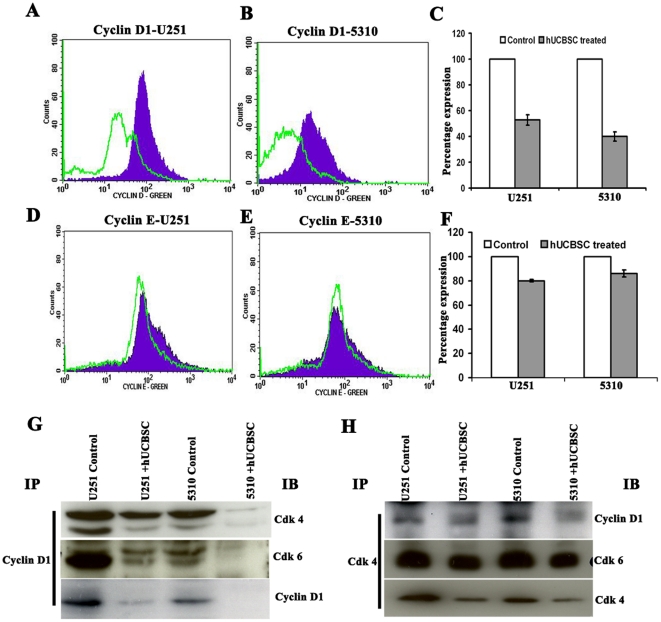
Flow cytometry analysis for expression of cyclins D1 and E. (**A**) and (**B**) Expression of cyclin D1 in U251 and 5310 alone and in co-cultures with hUCBSC as assessed by flow cytometry. Cyclins D1 and E expression was determined by staining with the mAb to human cyclin D1, E or isotype control mouse or rabbit IgG_1_, and then followed by a secondary antibody goat anti-mouse IgG or anti-rabbit IgG conjugated to Alexa Fluor green dye. The background has been subtracted using different isotypic controls for respective antibodies. (**D**) and (**E**) Expression of cyclin E in U251 and 5310 control cells and in co-cultures with hUCBSC. (**C**) and (**F**) Bar graphs representing the percent expression levels of cyclin D1 and E when compared with the normalized control. (**G**) Around 600 µg of total soluble protein was taken from U251 and 5310 control cells and hUCBSC-treated tissue lysates. The protein samples were incubated with 2 µg/ µl of Cdk 4 and cyclin D1 antibody. The mixture containing total soluble protein and respective antibody was incubated with 50 µL of protein A/G agarose beads for 30’ in ice. The Cdk 4-immunoprecipitated blot was immunoblotted against cyclin D1, Cdk 6 and Cdk 4, while the cyclin D1-immunoprecipitated blot was immunoblotted against Cdk 4, Cdk 6 and cyclin D1.

As assessed by flow cytometry, U251 and 5310 cells expressed cyclin D1 when compared to co-cultures and had higher mean fluorescence intensity, which is the calculated difference between the fluorescence intensity of the single and co-cultured cells. The pattern of cyclin D1 expression seen in the flow cytometry results suggest that cyclin D1-expressing cells reside mainly in the G_1_ phase of the cell cycle. The results obtained indicate that U251 cells, when co-cultured with hUCBSC showed 54% reduction, while co-cultured 5310 cells showed 34% reduction in cyclin D1 expression (Figs. 4A, 4B and 4C). Flow cytometric studies were conducted to study the expression levels of cyclin E in U251 and 5310 when co-cultured with hUCBSC. The results obtained demonstrate that hUCBSC reduce cyclin E expression levels in glioma cells after co-culture by only 20% in U251 and 14% in 5310 cells (Figs. 4D, 4E and 4F). Next, we conducted immuno precipitation experiments to study the effect of hUCBSC on cyclin D1, Cdk 4 and Cdk 4/Cdk 6 complexes. Cyclin D1 expression was observed to be reduced, when immunoprecipitated and immunoblotted with anti-cyclin D1 antibody. When hUCBSC treated cells were immunoprecipitated with cyclin D1 and immuno blotted with Cdk 4 and Cdk 6 antibodies, decrease in the expression levels of both Cdk 4 and Cdk 6 was observed (Fig. 4G). Similarly, when glioma cells alone and in co-culture with hUCBSC were immunoprecipitated with Cdk 4, immuno blotted with cyclin D1 and Cdk 6 antibodies, reduction was observed in the expression levels of both cyclin D1 and Cdk 6 (Fig. 4H), indicating that the hUCBSC treatment may not only reduce the individual expression of the respective genes, but also effect their complex formations, necessary for the transition from G_0_-G_1_ phase to other phases of the cell cycle.

### Downregulation of cyclin D1 by hUCBSC treatment in U251 and 5310 glioma nude mice models

**Figure 5:**
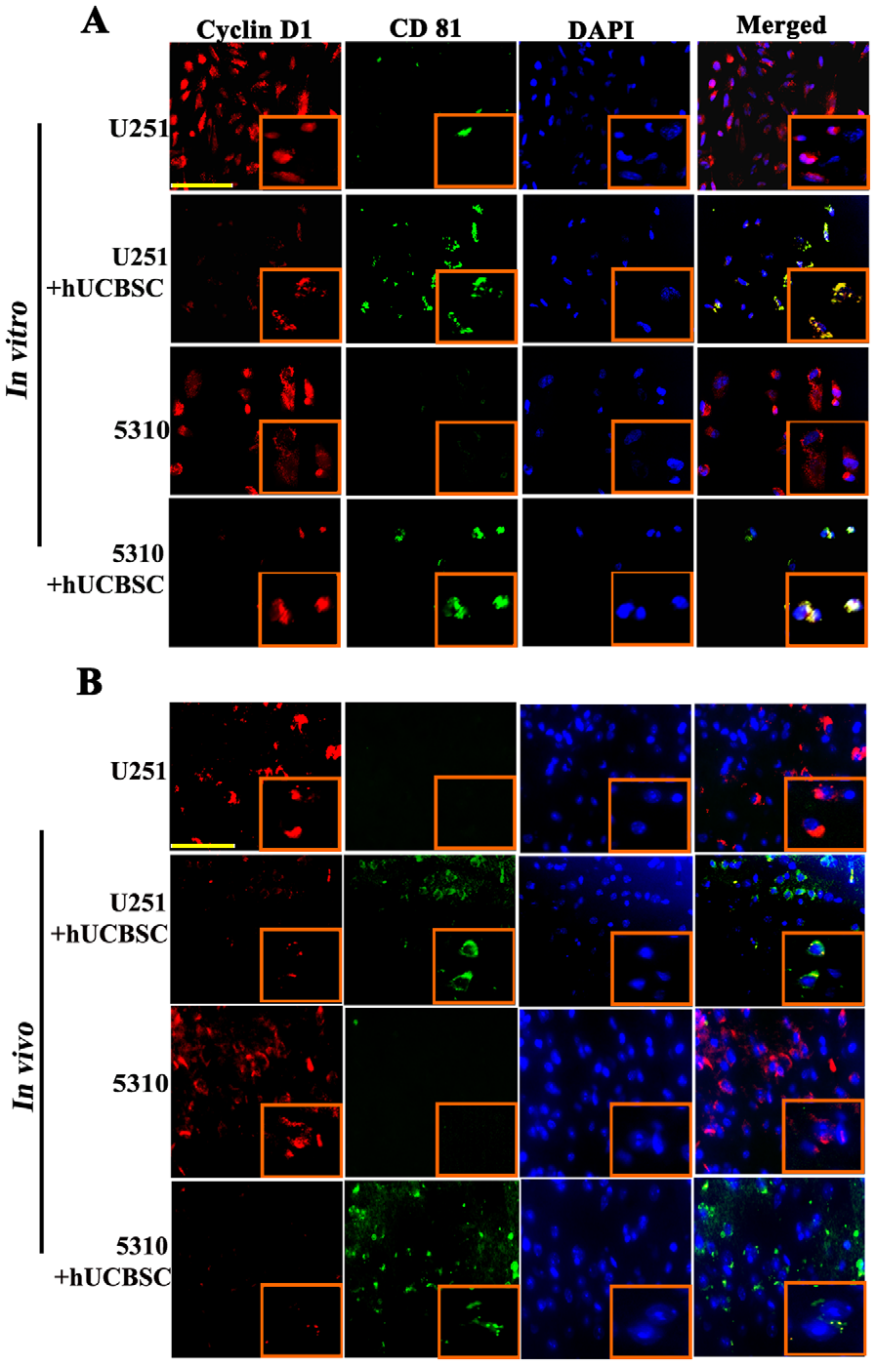
hUCBSC treatment downregulates cyclin D1 *in vitro* and *in vivo*. (**A**) Immunocytochemistry of single and co-cultures of U251 and 5310 cells for the expression of cyclin D1 and CD81. Cyclin D1 is conjugated with Alexa Fluor-594 (red), and CD81 is conjugated with Alexa Flour-488 (green). Inset pictures show magnified images. Bar = 200 µm (**B**) Nude mice with pre-established intracranial human glioma tumors (U251 or 5310) were treated with hUCBSC by intracranial injection (2×10^5^). Fourteen days after hUCBSC administration, the brains were harvested and sectioned and immunoprobed for cyclin D1 and appropriate secondary antibodies. Inset pictures show magnified images. Bar = 200 µm. Each experiment was performed in triplicates with each sample (n = 3).

To determine cyclin D1 expression after co-culture with hUCBSC *in vitro* and *in vivo* conditions, we carried out co-localization experiments with cyclin D1 and CD81 (mesenchymal stem cell marker for hUCBSC) antibodies. Glioma cells co-cultured with hUCBSC shows reduced expression of cyclin D1 in both cell lines (Fig. 5A). Similarly, tumor areas of xenografts showed reduced expression of cyclin D1 (Fig. 5B) in hUCBSC treated tumor sections when compared to the controls. Further, cyclin D1 was co-localized with CD81, confirming the presence of hUCBSC in treated brain samples. In both the cases, during co-localization of hUCBSC with glioma cells, it has been shown that hUCBSC should be in contact with the glioma cells to show their effect [24], [25]. It is plausible that hUCBSC reduced cyclin D1 expression in glioma cells within their contact and in the surrounding areas (Figs. 5A, 5B).

### hUCBSC down regulates the expression of cell cycle proteins at the transcriptional level

**Figure 6:**
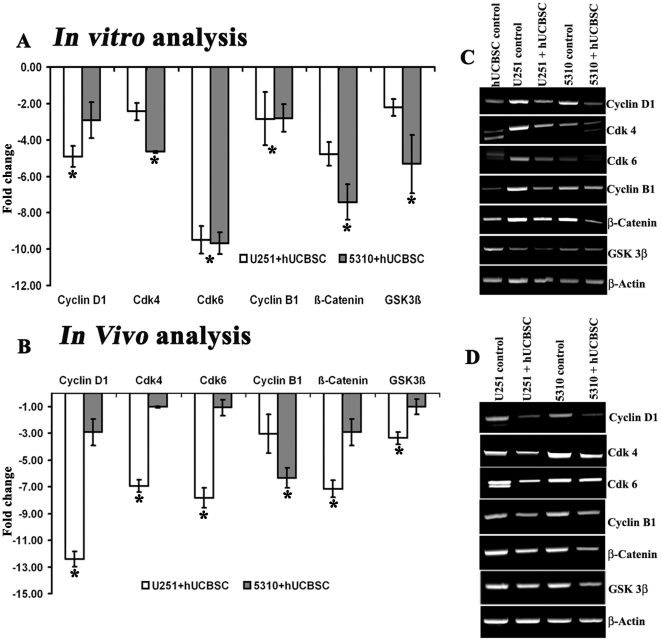
cDNA PCR microarray expression data and quantitative RT-PCR data for the differentially expressed genes *in vitro* and *in vivo.* (**A**) and (**B**) Genes downregulated in hUCBSC-treated U251 and 5310 cells and compared to control glioma cells. We used microarrays of cDNA clones selected from a cell cycle library to identify genes that were downregulated using the cDNA prepared from the above mentioned cell lysates. X-axis represents expression level in arbitrary units with the expressed genes on the y-axis. All the data presented here are from experiments performed in triplicate (n = 3). Expression of cyclin D1, cyclin B1, Cdk 4, Cdk 6, β-Catenin and Gsk-3β mRNAs under (C) *in vitro* and (D) *in vivo* conditions. β-actin is used as loading control. *Significant at p<0.05.

cDNA microarrays were done to determine and compare the native expression levels of various cell cycle proteins expressed in G_0_-G_1_, S, G_2_-M phases and other upstream molecules, in both glioma and their treatment with hUCBSC. Differential expression of cell cycle regulatory proteins such as cyclin D1, Cdk 4 and Cdk 6 were presented in Table 1. Our analysis showed that co-culture with hUCBSC has reduced the expression of cyclin D1 by -4.92 fold (*in vitro*) and -12.9 fold (*in vivo*) in U251, and −2.92 fold (*in vitro*) and -1.92 fold (*in vivo*) in 5310 cell lines, respectively (Figs. 6A, 6B). Semi-quantitative reverse transcriptase PCR results carried out on *in vitro* and *in vivo* samples showed reduced expression of cyclin D1, Cdk 4, Cdk 6 and cyclin B1 along with the upstream molecules β-Catenin and Gsk-3β (Figs. 6C, 6D).

**Table 1:** Differential expression of cell cycle proteins on treatment with hUCBSC.

Symbol	GenBankAccessionNo.	Description	Fold Change	
			5310+hUCBSC U251+hUCBSC	
CCND1	NM_053056	Cyclin D1	−2.3	−2.69
CCNB1	NM_031966	Cyclin B1	−1.3	−1.8
CCNE1	NM_001238	Cyclin E1	−1.6	−2.3
CDK4	NM_000075	Cyclin-dependent kinase 4	−1.9	−1.2
CDK6	NM_001259	Cyclin-dependent kinase 6	−3.5	−4.1

Human cell cycle cDNA arrays (SA Biosciences, Cat. No. PAHS-020) were run using cDNA from single and co-cultures of glioma cells with hUCBSC, as per manufacturer’s instructions as explained in Materials and Methods. Real time PCR was carried out and changes in gene expression were illustrated as fold increase/decrease according to manufacturer’s instructions. The cut-off induction determining expression was 2.0 or –2.0 fold changes. Genes that met these criteria were considered to be upregulated or downregulated.

## Discussion

Previously we have shown that hUCBSC plays an important role in inducing apoptosis [25], [26] and reduced migration [27] in glioblastoma cell lines. We focused our present study to determine the regulation of cell cycle progression in the glioma cells when co-cultured with hUCBSC and attempted to determine the potential of hUCBSC to mediate down regulation of cyclin D1, thereby reducing the cell cycle progression by arresting them at G_0_-G_1_ phase. Recent reports indicate that, increased expression of cyclin D1 leads to uncontrolled cell cycle in glioma biopsies; breast, head, neck, esophageal, rectal carcinomas; and astrocytoma cell lines [28]–[31]. Around 46.2% of ovarian carcinoma tissues were observed to have over expression of cyclin D1 associated with an ascending clinical stage and poor prognosis [32]. In addition to its function as a Cdk regulatory subunit and regulator of pRb, previous studies demonstrate that cyclin D1 also plays a role in cell differentiation [33]. Cyclin D1 is likely to be related to both tumor initiation and progression and may play a key role in promoting growth of glial cells and their transformation to malignant cells. A characteristic feature of cancer is uncontrolled cell division and disruption of a steady stage between apoptosis and cell division by loss of cell cycle control, which deregulates G_1_-S phase progression. Our current study was based on the hypothesis that uncontrolled glioma cell proliferation could be regulated by treating with human umbilical cord blood stem cells. In particular, we focused on the therapeutic aspects and potential of hUCBSC to control glioma cell proliferation by regulating the cell cycle. Previous studies have reported that mesenchymal stem cells may regulate cell cycle progression by upregulating the transcriptional expression of p21 and caspase-3, resulting in a G_0_-G_1_ phase arrest and apoptotic cell death of tumor cells [16].

Previously, we used multiple approaches including LDH release and MTT assays, to study the effect of hUCBSC when co-cultured with glioma cell lines [26]. In the present study, flow cytometry results indicated a significant reduction of cells in the G_0_-G_1_ phase of the cell division cycle, with hUCBSC treatment on co-cultures with U251 and 5310 cells. This is in agreement with our previous results, in which the rates of apoptosis were significantly increased when glioma cells were co-cultured with hUCBSC, indicating that hUCBSC induced apoptosis as a way to control the cell cycle progression of U251 and 5310 cells [26]. When hUCBSC alone were subjected to FACS analysis, the cells were in G_0_-G_1_ phase, indicating that the cells were in the resting stage. We observed that the stem cells remained in the quiescent stage until they received mitogenic stimuli. However, in co-cultures, hUCBSC arrested U251 and 5310 cells in the G_0_-G_1_ phase after 72 h of co-culture. These results prompted us to investigate the mechanism of action of hUCBSC on the cell cycle machinery. Western blot analysis of positive cell cycle regulators such as cyclin D1, Cdk 4, Cdk 6, and cyclin B1 showed a decrease in their expression levels, suggesting a G_0_-G_1_ arrest. As a positive regulator of Cdk 4 and Cdk 6, cyclin D1 has been implicated in controlling the G_1_ phase of the cell cycle as shown in human colon adenocarcinomas [34]. It has also been reported that over expression of an antisense cyclin D1 cDNA construct in a human colon carcinoma cell line leads to impaired cell growth and tumorigenicity, implying that cyclin D1 is an oncogene [35]. Recent evidences suggest that ras signaling can upregulate cyclin D1 and Cdk 4 assembly by sequential activation of ras, raf-1, ERK pathway [36]. Li et al (2010) reported that in U251 cells progression from the G_1_ to S phase of the cell cycle requires activation of Cdk4, which is controlled in part by complex formation with its catalytic partner, cyclin D1, which suggests that cyclin D1 is necessary for G1 progression. They have also observed that the occurrence of G_0_-G_1_ cell cycle arrest in U251 cells was consistent with down-regulation of cyclin D1 [37].

When quiescent cells are mitogen-stimulated, they enter the cell division cycle inducing transcription of the cyclin D1 gene [38]. When the mitogenic signal generated from upstream molecule is removed, it is postulated that transcription of the cyclin D1 gene stops, which results in a decline of cyclin D1 protein levels [38], [39] and indicates that cyclin D1 indeed acts as a regulator of cell cycle progression. Earlier reports from Etheridge *et al*., (2004) suggest that mesenchymal stem cells secrete Dickkopf-1 (Dkk-1) to suppress the Wnt/β-Catenin signaling pathway for attenuating the malignant phenotype of tumor cells [40]. Based on these earlier studies, we carried out additional experiments to study the expression of upstream molecules, including ERK, pERK and Gsk-3β, which may be instrumental in deporting the mitogenic signal to cyclin D1 and initiating cell cycle progression. Our results show that all these proteins were down regulated in U251 and 5310 cells co-cultured with hUCBSC. Cyclin D1 may probably be regulated upstream by the expression of β-catenin. The possible mechanism might be the activation of β-catenin by PI3k/AKT pathway as reported in HepG2 cells [41] and alveolar macrophages [42]. Earlier reports have shown that downregulation of β-catenin in osteosarcoma cells and experimental rat gliomas transfected with β-catenin siRNA decreases cyclin D1 expression levels. Our results revealed that treatment with hUCBSC not only decreased the expression of cyclin D1 but also that of β-catenin in U251 and 5310 cells as compared to the reported data in U251 glioblastoma cells [43], [44].

To study G_0_-G_1_ phase progression and the proteins expressed during this process, we targeted cyclin D1 and its partners, Cdk 4 and Cdk 6. We used fascaplysin, that specifically inhibits Cdk 4 and cyclin D1 complex, and thymidine, which synchronizes cells in the G_0_-G_1_ phase, to study the expression levels of cyclin D1, Cdk 4 and Cdk 6 and their roles in interrupting cell cycle progression [45]. Studies have demonstrated that fascaplysin arrested osteosarcoma cells U2OS, colon carcinoma cells HCT116 and diploid fibroblasts cells MRC-5 in G_1_ phase of cell cycle [45]. Moderate G_1_ arrest was observed using 4 mM Fascaplysin in BeL-7402, 2.6 mM in HUVEC [46]. Our studies conducted on U251 and 5310 cell lines using 1.0 µM Fascaplysin was found to inhibit their proliferation through inducing a G_1_ phase arrest demonstrating that the expression of cyclin D1 and Cdk 4 is indeed essential for the cells to complete its cell cycle normally.

We next sought to confirm that cyclin D1 expression was regulated in a normal way in the co-cultures with 4 mM thymidine [47]. To do so, we analyzed the cell cycle profile at various thymidine concentrations following after 12 h of treatment. Glioma cells proliferate rapidly in this condition whereas normal cells withdraw from the cell cycle. Cyclin D1 is reported to be one such protein that is intermittently overexpressed, thereby promoting uncontrolled cell proliferation [38]. With FACS and immunoblot analyses, we demonstrated that cyclin D1, Cdk 4 and Cdk 6 are essential for the cells to transit the G_0_-G_1_ phase and that cyclin D1 must be expressed to receive the mitogenic stimuli for the cells to initiate the cell cycle. Also, we found that when co-cultured with hUCBSC, glioma cells were mimicking the roles of both Fascaplysin and thymidine. Our results indicate that even though there is reduced expression of cyclin D1 and Cdk 4, the cells are still in G_0_-G_1_ phase, which shows that hUCBSC may use another pathway for its synchronization.

Earlier reports suggest that in the absence of cyclin D1 in cyclin D1 knockout mice, the levels of cyclin E1 were significantly higher, indicating its potential as a candidate molecule that stands in for cyclin D1 [48]. We next asked whether cyclin D1 could form a functional complex with its specific partners Cdk 4 and Cdk 6. We immunoprecipitated cyclin D1 and Cdk 4 and probed immunoblots with antibodies directed against Cdk 4, cyclin D1 or Cdk 6. These results demonstrate that Cdk 4 or Cdk 6 could be pulled down by either cyclin D1 or Cdk 4. Earlier studies conducted on several neoplasias suggest that cyclin E may mimic cyclin D1 by hyper-phosphorylating pRb in the absence of cyclin D1 [49]. cDNA PCR analysis done to elucidate the expression profiles of different genes, belonging to different cell cycle phases when co-cultured with hUCBSC have shown reduced expression. Thus, the role of hUCBSC in preventing cancer cell proliferation at the cellular level is evidenced by downregulation of expression of cyclin D1.

In conclusion, our results suggest hUCBSC can attenuate uncontrolled cell cycle progression, which is a hallmark of cancer, by down regulating the expression levels of cyclin D1 and its partner kinases Cdk 4 and Cdk 6 at the cell cycle level. We have also observed that despite the reduced expression of crucial cell cycle proteins such as cyclin D1, Cdk 4 and Cdk 6, we see an increased G_0_-G_1_ peak and our results from the present study confirm that this increase may be partially compensated by cyclin E expression. It is also confirmed that hUCBSC regulates the expression levels of cell cycle proteins and kinases both at the transcriptional and the translational levels. These results are in conformity with our previous results wherein we observed that intracranial glioma xenografts treated by hUCBSCs showed regression (24, 26). The present study paves the way for further research to better understand the role of the cyclin D1 pathway both at the cell cycle and molecular levels in stem cell-mediated glioma therapy. Studying the effects of reduced expression of cyclin D1 after treating with hUCBSC could potentially help us to understand the role of cyclin D1 in glioblastoma as well as, to further delineate the human glioblastomal hierarchy, reiterating the importance of hUCBSC in glioblastoma.

## Materials and Methods

### Ethics Statement

After obtaining informed consent, human umbilical cord blood was collected from healthy volunteers according to a protocol approved by the Peoria Institutional Review Board, Peoria, IL, USA. The consent was written and approved. The approved protocol number is 06-014, dated December 10, 2009. The Institutional Animal Care and Use Committee of the University Of Illinois College Of Medicine at Peoria, Peoria, IL, USA approved all surgical interventions and post-operative animal care. The consent was written and approved. The approved protocol number is 851, dated November 20, 2009.

### Antibodies and reagents

We used the following antibodies in this study: mouse anti-cyclin D1, mouse anti-cyclin E, rabbit anti-Cdk 4, mouse anti-Cdk 6, mouse anti-cyclin B1, mouse anti-p27, mouse anti-p21, mouse anti-β-Catenin, rabbit anti-Gsk-3β, rabbit anti-ERK, mouse anti-pERK, mouse anti-β-actin and mouse anti-GAPDH (1∶200; Santa Cruz Biotechnology Inc., Santa Cruz, CA). Fascaplysin chloride hydrate and thymidine were purchased from Sigma-Aldrich (St. Louis, MO).

### hUCBSC isolation and culture

Human umbilical cord blood stem cells were collected upon delivery from healthy donors with informed consent according to the protocol approved by the University of Illinois at Peoria Institutional Review Board. Human umbilical cord blood stem cells were isolated using Ficoll-Paque (GE Health Care, Piscataway, NJ) density gradient centrifugation [50]. The isolated cells were plated in 100 mm plates in DMEM knockout medium (Invitrogen, Carlsbad, CA) supplemented with 10% fetal bovine serum albumin, 10% knockout serum (Hyclone, Logan, UT), and 1% penicillin/streptomycin. Cells were allowed to adhere for 72 h and non-adherent cells were removed with medium changes. Once adherent cells reached approximately 20% to 30% confluence, cells were supplemented with Mesencult medium (Stem cell Technologies, Vancouver, Canada) supplemented with mesenchymal stem cell stimulatory supplements (Human) (Stem cell Technologies, Vancouver, Canada), 1% penicillin/streptomycin (Invitrogen, Carlsbad, CA) and plated in 100 mm culture dishes. Human astrocytes were purchased from Sciencell Research Laboratories (Carlsbad, CA) and were grown in astrocyte medium supplemented with 2% FBS, 1% penicillin-streptomycin and 1% astrocyte growth supplements (Sciencell Research Laboratories). For co-culture experiments, hUCBSC/astrocytes/rat bone marrow stromal cells and glioma cells were cultured at a ratio of 1∶4 (2.5×10^4^∶1×10^6^). Co-cultures of hUCBSC and U251 were grown in DMEM; co-cultures of hUCBSC and 5310 were grown in RPMI-1640; similarly co-cultures of astrocytes and U251 were grown in DMEM; co-cultures of astrocytes and 5310 were grown in RPMI-1640. For co-cultures with astrocytes, DMEM and RPMI-1640 were supplemented with 1% astrocyte growth supplements.

### Culture of rat bone marrow stromal cells

Rat primary mesenchymal stem cells isolated from the bone marrow of adult female Fisher 344 rats with markers integrin β1^+^ and CD54^+^ were obtained from Chemicon (Temecula, CA, USA) and maintained according to manufacturer’s instructions in DMEM-low glucose (Invitrogen, Carlsbad, CA, USA), supplemented with 10% heat-inactivated FBS (Hyclone, Logan, UT, USA), 2 mM L-Glutamine and 1% solution of Penicillin and Streptomycin (Invitrogen, Carlsbad, CA, USA). When cells reached 70–80% confluency, the cells were detached with TrypLE Express (Invitrogen, Carlsbad, CA, USA) and centrifuged at 250×g for 3 min, replated and maintained at 37°C in an incubator with a 5% CO_2_ atmosphere.

### Malignant glioma cell line culture conditions

U251 cells obtained from the National Cancer Institute (NCI)-Frederick (Frederick, MD) was grown in DMEM supplemented with 10% fetal bovine serum (FBS) (Hyclone, Logan, UT) and 1% penicillin-streptomycin (Invitrogen, Carlsbad, CA). Xenograft cell line 5310 (a kind gift from Dr. David James at University of California, San Francisco) was grown in RPMI-1640 medium supplemented with 10% FBS and 1% penicillin-streptomycin in a humidified atmosphere containing 5% CO_2_ at 37°C.

### RNA extraction and quantitative real time PCR

Primer sequences were designed from human GenBank sequences using Primer 3 software (v.0.4.0). For real time polymerase chain reaction (RT-PCR) analysis and RT-PCR-based microarray analysis (RT^2^ Profiler PCR Array, SuperArray, Frederick, MD), total cellular RNA was isolated from control and hUCBSC-treated cancer cells using the RNeasy kit (Qiagen, Valencia, CA) and quantified by measuring absorbance at 260 nm. Total RNA was reverse transcribed into first strand cDNA using the Transcriptor First Strand cDNA Synthesis Kit (Roche, Indianapolis, IN). PCR amplification for the specific genes was conducted for 30 cycles (94°C, 1 min; 60°C, 1 min; 72°C, 1 min). One-step SYBR green-based RT-PCR was optimized and carried out using the SYBR Green Quantitative RT-PCR Kit in the iCycler iQ Real-Time PCR Detection System (Bio-Rad). Amplification graphs were checked for the C_t_ value of the PCR product. The C_t_ value represents the cycle where the fluorescence of a sample increases to a level higher than the background fluorescence in the amplification cycle. Each sample was measured in triplicate and normalized with GAPDH or β-actin gene expression.

The following primers were used for real time PCR: Cyclin D1 (forward:5’gaggaagaggaggaggagga3’; reverse:5’gagatggaagggggaaagag3’); Cdk4 (forward: 5’gaaactctgaagccgaccag3’; reverse: 5’ggcagagattcgcttgtgt3’); Cdk6 (forward:5’gcacagtgtcacgaacaga3’; reverse: 5’cctcggagaagctgaaaca3’); Cyclin B1 (forward:5’ggccaaaatgcctatgaaga3’; reverse: 5’gatgtttccattgggcttg3’); β–Catenin (forward: 5’gaaacggctttcagttgag3’; reverse: 5’ctggccatatccaccagagt3’) and β-Actin (forward:5’ggcatcctcaccctgaagta3’; reverse: 5’ggggtgttgaaggtctcaaa3’).

### cDNA microarray analysis

For the present study, we used the Cell Cycle RT2 Profiler PCR Array (SuperArray Biosciences, Frederick, MD) because of its advantage in detecting the expression of several genes concurrently. Each array contains a panel of 96 primer sets of 84 pathway-focused genes along with five housekeeping genes and three RNA and PCR quality controls. The RT-PCR conditions for the one-step SYBR green RT-PCR consist of a one cycle of 94°C for 10 min, followed by 40 cycles of PCR at 95°C for 15 sec (denaturation), and 60°C for 1 min (annealing and extension). Data were analyzed using the SuperArray RT^2^ Profiler PCR Array Data Analysis Template (v3.0). Changes in gene expression were illustrated as a fold increase/decrease with 2.0 or –2.0 as the cut-off. Genes that met these criteria were considered to be either up- or downregulated. All of these experiments were performed in triplicate.

### Cell cycle analysis

Progression through different cell cycle phases was monitored by flow cytometric analysis of the DNA content of cell populations stained with propidium iodide and was carried out with a fluorescence-activated cell sorter (FACS Caliber flow cytometer; Becton Dickinson). The percentage of cells within G_1_, S, G_2_ and M phases was determined by using CellQuest software. Fascaplysin (1.0 µM) was added to the cultured glioma cells and incubated for 24 h and thymidine (4 mM) was incubated for 12 h prior to processing for FACS analysis. U251 and 5310 cells (2×10^5^) were seeded either alone or co-cultured with hUCBSC for 72 h. The cells were harvested and stained with propidium iodide (BioSure, Grass Valley, CA). Approximately, ten thousand events were counted for each analysis and 2 to 4 independent experiments performed in triplicate were conducted for each group.

To visualize two different populations of cells in the co-cultures by means of flow cytometry, glioma cell lines U251 and 5310, alone and in co-culture with hUCBSCs, were harvested after 72 h using 0.05% trypsin/EDTA (Invitrogen). Cells were gently disassociated into a single cell suspension and labeled with two different primary antibody surface markers at a 1∶100 dilution. Glioblastoma specific anti-rabbit glial fibrillary acidic protein (GFAP) and anti-goat CD81 for mesenchymal stem cells (hUCBSC) were used. After incubation at 37°C for 1 h, all cell combinations were washed and labeled with a corresponding secondary antibody conjugated to Alexafluor red (λ_594_) for GFAP and green (λ_488_) for CD81. Cells were subsequently washed twice in 1X phosphate buffered saline prior to FACS analysis. Cells were analyzed using the FACSCalibur flow cytometer (Becton Dickinson, San Jose, CA). Isotypic negative controls were used to establish background fluorescence. Positive cells were identified after excitation with the appropriate laser. The correct amp gain was determined using forward and side scatter to visualize the entire cell population. Dual color data were collected on 10,000 cells.

### Cyclin D1 and cyclin E1 expression as assessed by flow cytometry

Cyclin D1 and cyclin E1 expression was measured by intracellular protein staining using monoclonal antibodies for human cyclin D1 and cyclin E1 (Santa Cruz Technologies, Santa Cruz, CA). About 1×10^6^ U251 and 5310 cells alone or in co-cultures were fixed in 4% formaldehyde for 10 min. The cells were washed three times in phosphate buffered saline (PBS), resuspended in 0.1% Triton X-100 and incubated for 5 min. The cells were centrifuged for 5 min at 1000×*g* and blocked using 10% goat serum for 30 min. Later, the cells were incubated with gene-specific cyclin D1 or cyclin E1 for 1 h. The cells were washed with 1X PBS and incubated with Alexa fluor Green (λ_488_) for 30 min. The antibody was removed and the cells were washed three times with 1X PBS before processing for FACS analysis. The control for each sample was prepared identically, except an isotype-specific antibody was used instead of either the cyclin D1 or E1 antibody (mouse IgG_2_a). Cellular fluorescence was measured using the FACS Caliber flow cytometer (Becton Dickinson, San Jose, CA). Experiments were carried out in triplicates to ensure reproducibility of the results (n = 3).

### Immunoprecipitation of cyclin D1

Protein lysates were prepared from 3–5 mm^3^ pieces of frozen intracranial tumors. Approximately 200–400 µg of protein cell lysates were incubated with 50 µL of Protein G/A beads (Miltenyi Biotec, Auburn, CA) and followed by sequential additions of 10 µL (2 µg) of cyclin D1 and Cdk 4 antibodies with end-to-end rotation overnight at 4°C. The immunoprecipitates were then loaded onto ‘ µ’ columns (Miltenyi Biotec), and rinsed with lysis buffer. The columns were washed twice with 200 µL of lysis buffer. Pre-heated (95°C) 1x SDS gel loading buffer was loaded onto the column matrix using a fresh pipette tip and was incubated for 5 min at room temperature. After discharging the supernatant, 50 µL of 1x SDS gel loading buffer was added to the immunoprecipitates, and the supernatants were then collected and loaded into 10–12% SDS-PAGE, followed by electrophoretic transfer to nitrocellulose membranes for further analysis.

### Western blot analysis

Single and co-cultures of glioma cells or nude mice brain tissues were harvested and homogenized in four volumes of homogenization buffer [pH 7.4; 250 mM sucrose, 10 mM HEPES, 10 mM Tris-HCl, 10 mM KCl, 1% NP-40, 1 mM NaF, 1 mM Na3VO4, 1 mM EDTA, 1 mM DTT and 0.5 mM PMSF plus protease inhibitors (1 µg/mL pepstatin, 10 µg/mL leupeptin and 10 µg/mL aprotinin)] using a Teflon-fitted glass homogenizer. The homogenate was centrifuged at 13,000x*g* for 15 min at 4°C, and the protein levels in the supernatant were determined using the BCA assay (Pierce, Rockford, IL). Samples (40–50 µg of total protein/well) were subjected to 10–14% SDS-PAGE and transferred onto nitrocellulose membranes. The reaction was detected using Hyperfilm-MP autoradiography film (Amersham, Piscataway, NJ). Experiments were performed in triplicate.

### Intracranial tumor growth

The protocol, which was approved by the Institutional Animal Care and Use Committee of the University Of Illinois College Of Medicine at Peoria (Peoria, IL, USA), was followed for surgical interventions and post-operative animal care. U251 cells (1×10^6^) and 5310 cells (8×10^5^) were intracerebrally injected into the right side of the brain of nude mice. hUCBSC were injected near the left side of the brain after a week of tumor implantation. The ratio of the hUCBSC to cancer cells was maintained at 1∶4. Three weeks after tumor inoculation, six mice from each group were sacrificed by cardiac perfusion with 4% formaldehyde in PBS. The isolated brains were paraffinized and visualized with H & E staining [24].

### Immunohistochemistry of cyclin D1 expression in tumor sections

For cyclin D1 immunostaining, we used mouse monoclonal antibody specific for cyclin D1 at a 1∶100 dilution. For each case, 6 µ-thick sections were cut from the paraffin blocks. The sections from control and hUCBSC-treated mice brains were deparaffinized, rehydrated and blocked in 10% goat serum for an hour. Sections were incubated in the primary antibody solution overnight at 4°C in a humidified chamber and then washed in 1X PBS, incubated with the appropriate secondary antibody for 1 h, visualized and analyzed using a confocal microscope. For immunofluorescence, sections were treated with primary antibodies overnight at 4°C and then treated with appropriate Alexa fluor secondary antibodies at room temperature for 1 h. Negative controls were maintained either without primary antibody or using IgG isotype. Antigen retrieval was performed in the citrate buffer (pH 6.0). All immuno-stained sections when stained with DAB were counterstained with hematoxylin, and sections stained with fluorescent antibodies were counterstained with DAPI. For negative controls, the immune serum was replaced either with PBS or non-immune serum. The sections were blind reviewed by a neuropathologist.

### Statistical analysis

Quantitative data from cell counts, FACS analysis, western blot analysis, and other assays were evaluated for statistical significance using one-way analysis of variance (ANOVA). Data for each treatment group were represented as mean ± SEM and compared with other groups for significance by one way ANOVA using Graph Pad Prism version 3.02, a statistical software package.

## Supporting Information

Figure S1 **To characterize hUCBSC, we used (A) CD81 and (B) CD29 markers.** Stem cells were probed with anti-goat CD81 and anti-mouse CD29 antibodies and processed for FACS analysis. Control hUCBSC cells were used as negative controls. All the data presented here are from experiments performed in triplicate (n = 3).
(TIF)

Figure S2 **U251 cells in co-culture with hUCBSCs, were harvested after 72 h using 0.05% trypsin/EDTA.** Cells were gently disassociated into a single cell suspension and labeled with two different primary antibody surface markers at a 1∶100 dilution. Glioblastoma specific anti-rabbit glial fibrillary acidic protein (GFAP) and anti-goat CD81 for mesenchymal stem cells (hUCBSC) were used. After incubation at 37°C for 1 h, co-cultures were washed and labeled with a corresponding secondary antibody conjugated to Alexa fluor red (λ_594_) for GFAP and green (λ_488_) for CD81. Cells were subsequently washed twice in 1X PBS prior to FACS analysis. Cells were analyzed using the FACSCalibur flow cytometer. Isotypic negative controls were used to establish background fluorescence. Positive cells were identified after excitation with the appropriate laser. Dual color data were collected on more than 10,000 cells. n = 3.
(TIF)

Figure S3 **5310 cells in co-culture with hUCBSCs, were harvested after 72 h using 0.05% trypsin/EDTA.** Cells were gently disassociated into a single cell suspension and labeled with two different primary antibody surface markers at a 1∶100 dilution. Glioblastoma specific anti-rabbit glial fibrillary acidic protein (GFAP) and anti-goat CD81 for mesenchymal stem cells (hUCBSC) were used. After incubation at 37°C for 1 h, co-cultures were washed and labeled with a corresponding secondary antibody conjugated to Alexa fluor red (λ_594_) for GFAP and green (λ_488_) for CD81. Cells were subsequently washed twice in 1X PBS prior to FACS analysis. Cells were analyzed using the FACSCalibur flow cytometer. Isotypic negative controls were used to establish background fluorescence. Positive cells were identified after excitation with the appropriate laser. Dual color data were collected on more than 10,000 cells. n = 3.
(TIF)

Figure S4 (**A**) Western analysis for ERK, pERK, GSK-3β was done with U251, 5310 and their respective co-cultures with hUCBSC. All the data presented here are from experiments performed in triplicate (n = 3). (B) Single and co-cultures of glioma cells with astrocytes. Approximately, 40 µg of total protein lysate were loaded onto 12% gels and transferred onto nitrocellulose membranes and probed with respective antibodies. Immunoreactive bands were visualized using chemiluminescence ECL western blotting detection reagents and the reaction was detected using Hyperfilm-MP autoradiography film. GAPDH is served as the loading control. Each experiment was repeated three times.
(TIF)

## References

[1] Walker AE, Robins M, Weinfeld FD (1985). Epidemiology of brain tumors: the national survey of intracranial
neoplasms.. Neurology.

[2] Surawicz TS, Davis F, Freels S, Laws ER, Menck HR (1998). Brain tumor survival: results from the National Cancer Data
Base.. J Neurooncol.

[3] Donato V, Papaleo A, Castrichino A, Banelli E, Giangaspero F (2007). Prognostic implication of clinical and pathologic features in
patients with glioblastoma multiforme treated with concomitant radiation
plus temozolomide.. Tumori.

[4] Kang SG, Kim JH, Nam DH, Park K (2005). Clinical and radiological prognostic factors of anaplastic
oligodendroglioma treated by combined therapy.. Neurol Med Chir (Tokyo).

[5] Stupp R, Mason WP, van den Bent MJ, Weller M, Fisher B (2005). Radiotherapy plus concomitant and adjuvant temozolomide for
glioblastoma.. N Engl J Med.

[6] Lang FF, Bruner JM, Fuller GN, Aldape K, Prados MD (2003). Phase I trial of adenovirus-mediated p53 gene therapy for
recurrent glioma: biological and clinical results.. J Clin Oncol.

[7] Aboody KS, Brown A, Rainov NG, Bower KA, Liu S (2000). Neural stem cells display extensive tropism for pathology in
adult brain: evidence from intracranial gliomas.. Proc Natl Acad Sci USA.

[8] Benedetti S, Pirola B, Pollo B, Magrassi L, Bruzzone MG (2000). Gene therapy of experimental brain tumors using neural progenitor
cells.. Nat Med.

[9] Ehtesham M, Kabos P, Kabosova A, Neuman T, Black KL (2002). The use of interleukin 12-secreting neural stem cells for the
treatment of intracranial glioma.. Cancer Res.

[10] Ehtesham M, Kabos P, Gutierrez MA, Chung NH, Griffith TS (2002). Induction of glioblastoma apoptosis using neural stem
cell-mediated delivery of tumor necrosis factor-related apoptosis-inducing
ligand.. Cancer Res.

[11] Caplan AI, Bruder SP (2001). Mesenchymal stem cells: building blocks for molecular medicine in
the 21st century.. Trends Mol Med.

[12] Tocci A, Forte L (2003). Mesenchymal stem cell: use and perspectives.. Hematol J.

[13] Wang JC, Doedens M, Dick JE (1997). Primitive human hematopoietic cells are enriched in cord blood
compared with adult bone marrow or mobilized peripheral blood as measured by
the quantitative in vivo SCID-repopulating cell assay.. Blood.

[14] Williams B, Allan DJ (1996). Combination of SCF, IL-6, IL-3, and GM-CSF increases the mitotic
index in short term bone marrow cultures from acute promyelocytic leukemia
(APL) patients.. Cancer Genet Cytogenet.

[15] Feng B, Chen L (2009). Review of mesenchymal stem cells and tumors: executioner or
coconspirator?. Cancer Biother Radiopharm.

[16] Lu YR, Yuan Y, Wang XJ, Wei LL, Chen YN (2008). The growth inhibitory effect of mesenchymal stem cells on tumor
cells in vitro and in vivo.. Cancer Biol Ther.

[17] Fu M, Wang C, Li Z, Sakamaki T, Pestell RG (2004). Minireview: Cyclin D1: normal and abnormal
functions.. Endocrinology.

[18] Gillett C, Smith P, Gregory W, Richards M, Millis R (1996). Cyclin D1 and prognosis in human breast cancer.. Int J Cancer.

[19] Hall M, Peters G (1996). Genetic alterations of cyclins, cyclin-dependent kinases, and Cdk
inhibitors in human cancer.. Adv Cancer Res.

[20] Molenaar JJ, Ebus ME, Koster J, van SP, van Noesel CJ (2008). Cyclin D1 and CDK4 activity contribute to the undifferentiated
phenotype in neuroblastoma.. Cancer Res.

[21] James CG, Woods A, Underhill TM, Beier F (2006). The transcription factor ATF3 is upregulated during chondrocyte
differentiation and represses cyclin D1 and A gene
transcription.. BMC Mol Biol.

[22] Mejlvang J, Kriajevska M, Vandewalle C, Chernova T, Sayan AE (2007). Direct repression of cyclin D1 by SIP1 attenuates cell cycle
progression in cells undergoing an epithelial mesenchymal
transition.. Mol Biol Cell.

[23] Takahashi M, Kojima M, Nakajima K, Suzuki-Migishima R, Takeuchi T (2007). Functions of a jumonji-cyclin D1 pathway in the coordination of
cell cycle exit and migration during neurogenesis in the mouse
hindbrain.. Dev Biol.

[24] Guo Y, Yang K, Harwalkar J, Nye JM, Mason DR (2005). Phosphorylation of cyclin D1 at Thr 286 during S phase leads to
its proteasomal degradation and allows efficient DNA
synthesis.. Oncogene.

[25] Gondi CS, Gogineni VR, Chetty C, Dasari VR, Gorantla B (2010). Induction of apoptosis in glioma cells requires cell-to-cell
contact with human umbilical cord blood stem cells.. Int J Oncol.

[26] Dasari VR, Velpula KK, Kaur K, Fassett D, Klopfenstein JD (2010). Cord Blood Stem Cell-Mediated Induction of Apoptosis in Glioma
Downregulates X-Linked Inhibitor of Apoptosis Protein
(XIAP).. PLoS One.

[27] Dasari VR, Kaur K, Velpula KK, Gujrati M, Fassett D (2010). Upregulation of PTEN in glioma cells by cord blood mesenchymal
stem cells inhibits migration via downregulation of the PI3K/Akt
pathway.. PLoS One.

[28] Buschges R, Weber RG, Actor B, Lichter P, Collins VP (1999). Amplification and expression of cyclin D genes (CCND1, CCND2 and
CCND3) in human malignant gliomas.. Brain Pathol.

[29] Jeon YT, Kim JW, Song JH, Park NH, Song YS (2005). Cyclin D1 G870A polymorphism and squamous cell carcinoma of the
uterine cervix in Korean women.. Cancer Lett.

[30] Kong S, Amos CI, Luthra R, Lynch PM, Levin B (2000). Effects of cyclin D1 polymorphism on age of onset of hereditary
nonpolyposis colorectal cancer.. Cancer Res.

[31] Monteiro E, Varzim G, Pires AM, Teixeira M, Lopes C (2004). Cyclin D1 A870G polymorphism and amplification in laryngeal
squamous cell carcinoma: implications of tumor localization and tobacco
exposure.. Cancer Detect Prev.

[32] Wu GQ, Xie D, Yang GF, Liao YJ, Mai SJ (2009). Cell cycle-related kinase supports ovarian carcinoma cell
proliferation via regulation of cyclin D1 and is a predictor of outcome in
patients with ovarian carcinoma.. Int J Cancer.

[33] Gillett C, Fantl V, Smith R, Fisher C, Bartek J (1994). Amplification and overexpression of cyclin D1 in breast cancer
detected by immunohistochemical staining.. Cancer Res.

[34] Bartkova J, Lukas J, Strauss M, Bartek J (1994). The PRAD-1/cyclin D1 oncogene product accumulates aberrantly in a
subset of colorectal carcinomas.. Int J Cancer.

[35] Arber N, Doki Y, Han EK, Sgambato A, Zhou P (1997). Antisense to cyclin D1 inhibits the growth and tumorigenicity of
human colon cancer cells.. Cancer Res.

[36] Cheng M, Sexl V, Sherr CJ, Roussel MF (1998). Assembly of cyclin D-dependent kinase and titration of p27Kip1
regulated by mitogen-activated protein kinase kinase (MEK1).. Proc Natl Acad Sci U S A.

[37] Li G, Wang R, Gao J, Deng K, Wei J (2010). RNA interference-mediated silencing of iASPP induces cell
proliferation inhibition and G0/G1 cell cycle arrest in U251 human
glioblastoma cells.. Molecular Cell Biochem.

[38] Sherr CJ (1996). Cancer cell cycles.. Science.

[39] Han L, Yang Y, Yue X, Huang K, Liu X (2010). Inactivation of PI3K/AKT signaling inhibits glioma cell growth
through modulation of β-catenin-mediated transcription.. Brain Res.

[40] Etheridge SL, Spencer GJ, Heath DJ, Genever PG (2004). Expression profiling and functional analysis of Wnt signaling
mechanisms in mesenchymal stem cells.. Stem Cells.

[41] Desbois-Mouthon C, Cadoret A, Blivet-Van Eggelpoel MJ, Bertrand F, Cherqui G (2001). Insulin and IGF-1 stimulate the beta-catenin pathway through two
signaling cascades involving GSK-3beta inhibition and Ras
activation.. Oncogene.

[42] Monick MM, Carter AB, Robeff PK, Flaherty DM, Peterson MW (2001). Lipopolysaccharide activates Akt in human alveolar macrophages
resulting in nuclear accumulation and transcriptional activity of
beta-catenin.. J Immunol.

[43] Guo Y, Harwalkar J, Stacey DW, Hitomi M (2005a). Destabilization of cyclin D1 message plays a critical role in
cell cycle exit upon mitogen withdrawal.. Oncogene.

[44] Pu P, Zhang Z, Kang C, Jiang R, Jia Z (2009). Downregulation of Wnt2 and beta-catenin by siRNA suppresses
malignant glioma cell growth.. Cancer Gene Ther.

[45] Takayama S, Rogatsky I, Schwarcz LE, Darimont BD (2006). The glucocorticoid receptor represses cyclin D1 by targeting the
Tcf-beta-catenin complex.. J Biol Chem.

[46] Soni R, Muller L, Furet P, Schoepfer J, Stephan C (2000). Inhibition of cyclin-dependent kinase 4 (Cdk4) by fascaplysin, a
marine natural product.. Biochem Biophys Res Commun.

[47] Zheng YL, Lu XL, Lin J, Chen HM, Yan XJ (2009). Direct effects of fascaplysin on human umbilical vein endothelial
cells attributing the anti-angiogenesis activity.. Biomed Pharmacother.

[48] Chen Z, Duan RS, Zhu Y, Folkesson R, Albanese C (2005). Increased cyclin E expression may obviate the role of cyclin D1
during brain development in cyclin D1 knockout mice.. J Neurochem.

[49] Bowe DB, Kenney NJ, Adereth Y, Maroulakou IG (2002). Suppression of Neu-induced mammary tumor growth in cyclin D1
deficient mice is compensated for by cyclin E.. Oncogene.

[50] Dasari VR, Veeravalli KK, Saving KL, Gujrati M, Klopfenstein JD (2008). Neuroprotection by cord blood stem cells against
glutamate-induced apoptosis is mediated by Akt pathway.. Neurobiol Dis.

