# Expression of Concern: Regulation of Glioblastoma Progression by Cord Blood Stem Cells Is Mediated by Downregulation of Cyclin D1

**DOI:** 10.1371/journal.pone.0337964

**Published:** 2025-12-02

**Authors:** The PLOS One Editors

Following the publication of this article [1], concerns were raised that the Fig S4A GAPDH panel of this article [1] appears similar to the Fig 5A and Fig 5B GAPDH panels of [2, retracted in 3], the Fig 5D and left Fig S3 GAPDH panels of [4], retracted in [5], and the Fig 7C GAPDH panel of [6, retracted in 7], despite being used to represent different experimental conditions.

The authors did not respond to editorial communications regarding these concerns.

In light of the concerns raised the *PLOS One* Editors issue this Expression of Concern to notify readers of the concerns with Fig S4 and to inform readers that these results should be interpreted with caution.
