# Supplementary figures and images for: Regulation of Glioblastoma Progression by Cord Blood Stem Cells Is Mediated by Downregulation of Cyclin D1
Source: PLoS One. 2011 Mar 24;6(3):e18017. doi: 10.1371/journal.pone.0018017 (PMC3063796; doi:10.1371/journal.pone.0018017)

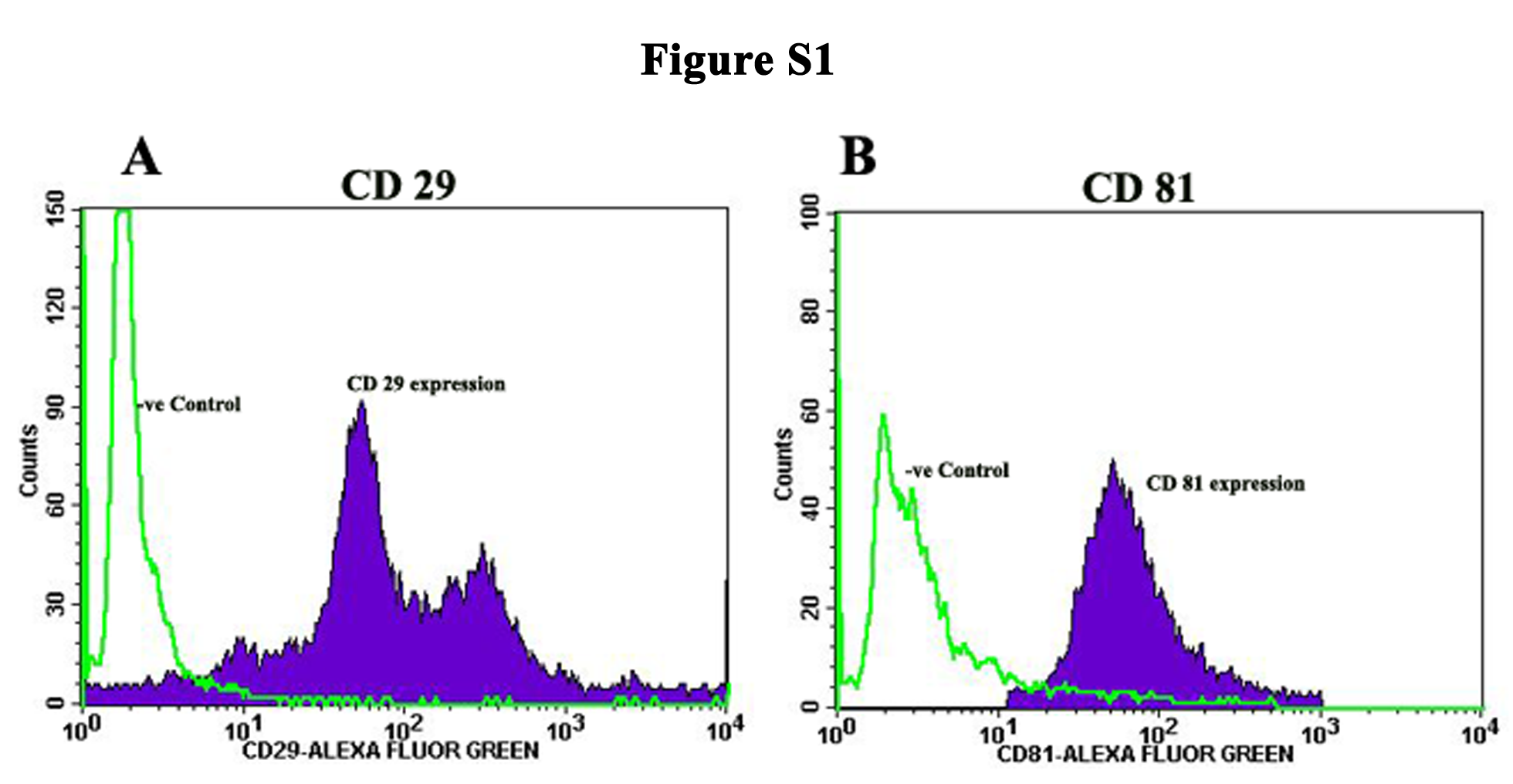

Supplement: Figure S1 — To characterize hUCBSC, we used (A) CD81 and (B) CD29 markers. Stem cells were probed with anti-goat CD81 and anti-mouse CD29 antibodies and processed for FACS analysis. Control hUCBSC cells were used as negative controls. All the data presented here are from experiments performed in triplicate (n = 3). (TIF) [file pone.0018017.s001.tif]

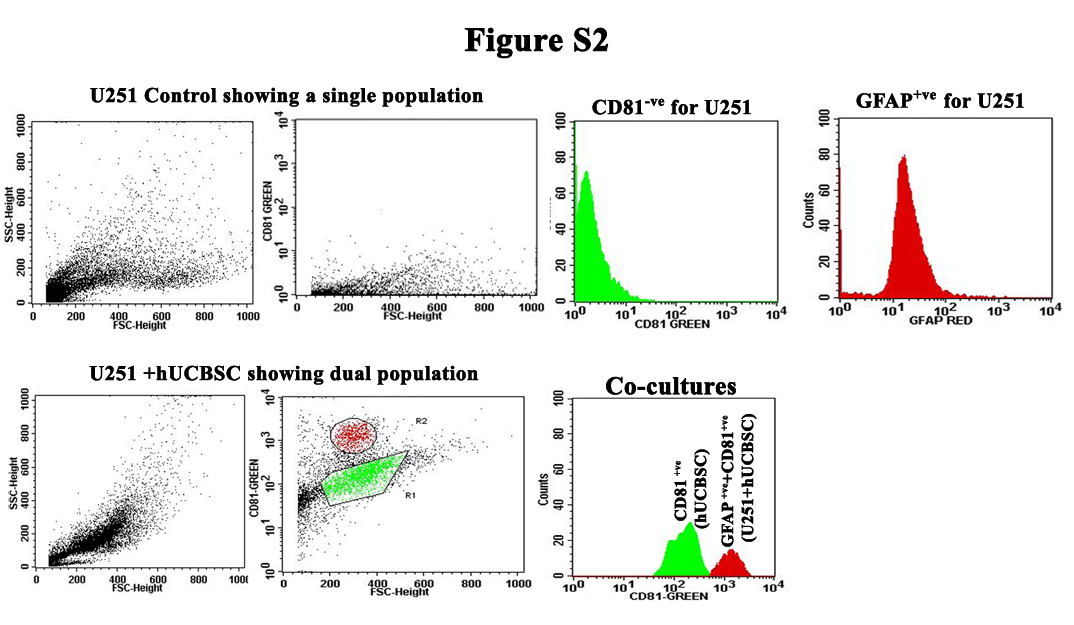

Supplement: Figure S2 — U251 cells in co-culture with hUCBSCs, were harvested after 72 h using 0.05% trypsin/EDTA. Cells were gently disassociated into a single cell suspension and labeled with two different primary antibody surface markers at a 1∶100 dilution. Glioblastoma specific anti-rabbit glial fibrillary acidic protein (GFAP) and anti-goat CD81 for mesenchymal stem cells (hUCBSC) were used. After incubation at 37°C for 1 h, co-cultures were washed and labeled with a corresponding secondary antibody conjugated to Alexa fluor red (λ594) for GFAP and green (λ488) for CD81. Cells were subsequently washed twice in 1X PBS prior to FACS analysis. Cells were analyzed using the FACSCalibur flow cytometer. Isotypic negative controls were used to establish background fluorescence. Positive cells were identified after excitation with the appropriate laser. Dual color data were collected on more than 10,000 cells. n = 3. (TIF) [file pone.0018017.s002.tif]

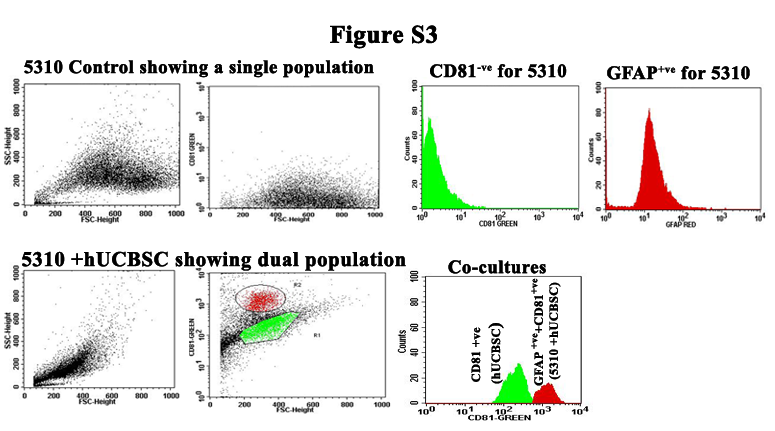

Supplement: Figure S3 — 5310 cells in co-culture with hUCBSCs, were harvested after 72 h using 0.05% trypsin/EDTA. Cells were gently disassociated into a single cell suspension and labeled with two different primary antibody surface markers at a 1∶100 dilution. Glioblastoma specific anti-rabbit glial fibrillary acidic protein (GFAP) and anti-goat CD81 for mesenchymal stem cells (hUCBSC) were used. After incubation at 37°C for 1 h, co-cultures were washed and labeled with a corresponding secondary antibody conjugated to Alexa fluor red (λ594) for GFAP and green (λ488) for CD81. Cells were subsequently washed twice in 1X PBS prior to FACS analysis. Cells were analyzed using the FACSCalibur flow cytometer. Isotypic negative controls were used to establish background fluorescence. Positive cells were identified after excitation with the appropriate laser. Dual color data were collected on more than 10,000 cells. n = 3. (TIF) [file pone.0018017.s003.tif]

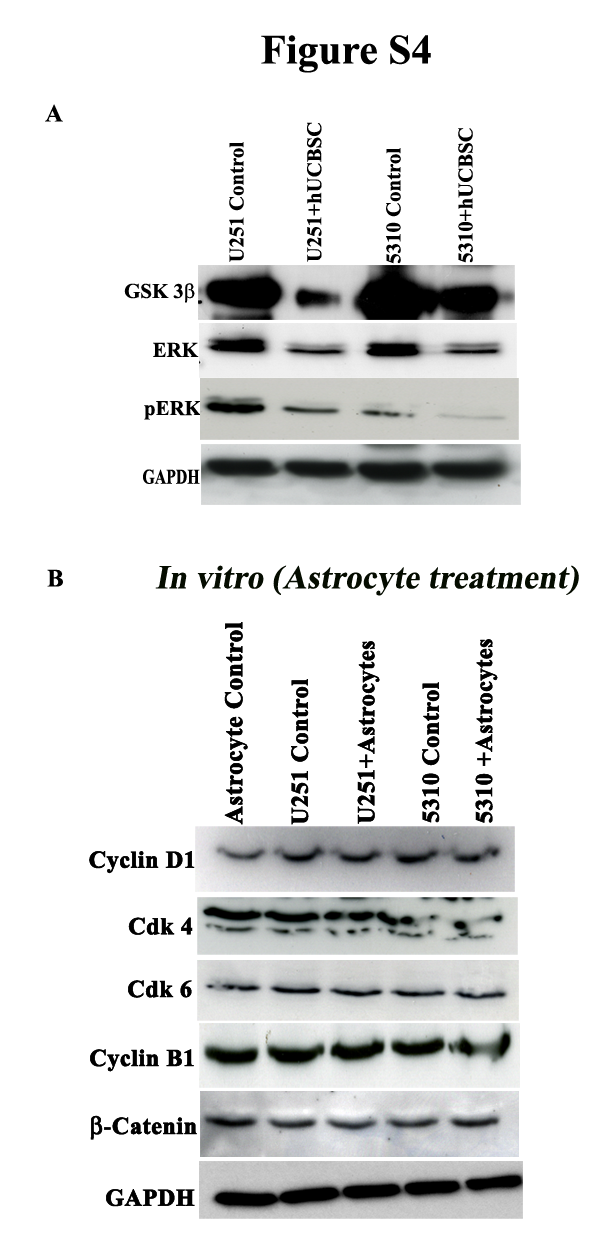

Supplement: Figure S4 — (A) Western analysis for ERK, pERK, GSK-3β was done with U251, 5310 and their respective co-cultures with hUCBSC. All the data presented here are from experiments performed in triplicate (n = 3). (B) Single and co-cultures of glioma cells with astrocytes. Approximately, 40 µg of total protein lysate were loaded onto 12% gels and transferred onto nitrocellulose membranes and probed with respective antibodies. Immunoreactive bands were visualized using chemiluminescence ECL western blotting detection reagents and the reaction was detected using Hyperfilm-MP autoradiography film. GAPDH is served as the loading control. Each experiment was repeated three times. (TIF) [file pone.0018017.s004.tif]
